# What Stops Fairness from Emerging in Assessment? The Forces on a Complex Adaptive System

**DOI:** 10.5334/pme.994

**Published:** 2023-08-24

**Authors:** Nyoli Valentine, Steven J. Durning, Ernst Michael Shanahan, Lambert Schuwirth

**Affiliations:** 1 Prideaux Discipline of Clinical Education, Flinders University, Bedford Park, South Australia, Australia; 2 Center for Health Professions Education, Uniformed Services University of the Health Sciences, Bethesda, Maryland, USA; 3 Flinders University, Bedford Park, South Australia, Australia

## Abstract

**Introduction:**

Workplace-based assessment occurs in authentic, dynamic clinical environments where reproducible, measurement-based assessments can often not be implemented. In these environments, research approaches that respect these multiple dynamic interactions, such as complexity perspectives, are encouraged. Previous research has shown that fairness in assessment is a nonlinear phenomenon that emerges from interactions between its components and behaves like a complex adaptative system. The aim of this study was to understand the external forces on the complex adaptive system which may disrupt fairness from emerging.

**Methods:**

We conducted online focus groups with a purposeful sample of nineteen academic leaders in the Netherlands. We used an iterative approach to collection, analysis and coding of the data and interpreted the results using a lens of complexity, focusing on how individual elements of fairness work in concert to create systems with complex behaviour.

**Results:**

We identified three themes of forces which can disrupt fairness: forces impairing interactivity, forces impairing adaption and forces impairing embeddedness. Within each of these themes, we identified subthemes: assessor and student forces, tool forces and system forces.

**Discussion:**

Consistent with complexity theory, this study suggests there are multiple forces which can hamper the emergence of fairness. Whilst complexity thinking does not reduce the scale of the challenge, viewing forces through this lens provides insight into why and how these forces are disrupting fairness. This allows for more purposeful, meaningful changes to support the use of fair judgement in assessment in dynamic authentic clinical workplaces.

## Introduction

Workplace-based assessment affords learners the opportunity to experience and overcome the real-life challenges clinicians face in delivering patient care. However, this level of ‘authenticity’ can create significant challenges for assessment. For example, time pressured clinical situations, uncontrolled encounters and the prioritisation of patient care all make standardised workplace assessment challenging. Different strategies have been used to attempt to overcome these problems such as the use of programmes of assessment [1], improved understanding of sampling and validity evidence [2], the use of entrustment decisions [3] and narrative approaches [4]. However, the realities of the healthcare environment means that despite the best intentions, assessment may occur in an unpredictable manner. Despite these challenges, assessment and learning still occur. Medical students become future health care professionals. As Greenhalgh notes of health services research, “the articulations, workarounds and muddling-through that keep the show on the road are not footnotes in the story but its central plot [5].” This also is true of medical education.

A lens of complexity has been encouraged to comprehend the nature of health professions education [6,7,8,9,10] because the environments in which assessment occurs are dynamic with numerous complex relationships and contexts. Linear rules or algorithms cannot be applied to every possible situation. Thus, to offer an understanding of this “muddling-through” [5], considering assessment as a complex adaptive system (CAS), which has significant explanatory power, is warranted. A CAS is a collection of individual agents with freedom to act in ways that are not always predictable, and whose actions are interconnects so that one agent’s actions change the context for the other agents [11]. The key features of a CAS are described in Table 1.

**Table 1:** Key features of a complex adaptive system (CAS).


**Independence:**	CAS consist of individual agents [11] who make independent choices about their actions [8].

**Adaptability:**	Each agent adapts to changes in the context, past experience and to each other’s behaviour [6,7,8,12].

**Unpredictability:**	The independence and adaptability of the agents leads to non-linearity and unpredictability [5,6,9,12].

**Emergence:**	Interactions between agents create outcomes that are greater than the sum of the individual agent behaviours [6]. The system’s behaviour relies less on the nature of the individual agents than on the quantity and quality of connections between them [8].

**Patterns:**	Despite the unpredictability, principles and patterns arise [12]. These patterns provide understanding to how the system works [9] as they guide behaviours within it [12].

**Distributed control:**	Control is dispersed as a result of a huge number of decisions made by individual agents [13] making the system resistent to centralised control [6,14].

**Self-organisation:**	Order, innovation and progress naturally arise from within the system [5,15]. Work arounds and muddling through are central to CAS [5,8]. Tensions and paradoxes do not necessarily need to be resolved [11].

**Embeddedness:**	Agents and CASs are embedded within other CASs [6]. Therefore, agents or systems cannot be understood without reference to the other systems [11,14].

**Fuzzy, ill-defined boundaries:**	The system boundaries are permeable and hard to define [6].


One specific area in which a complexity perspective may provide plausible insights is understanding the nature of fair judgements in assessment. Traditionally, fairness has been seen as synonymous with objectivity, however, measurement-based assessment has struggled to adequately evaluate the wide variety of competencies demanded of today’s health professionals. This has led to an increasing push to embrace human judgement in assessment and accept its subjective nature [2,16,17,18,19,20,21,22,23]. But to do this, we need to understand ‘What makes human judgement fair?.’

Unfortunately, there is no simple definition of fairness. It has been noted that fairness is a multi-faceted construct, with many different interacting components [24,25,26]. Recently, researchers identified a fractal suggesting fairness behaves as a CAS rather than a linear process, and that it emerges from numerous dynamic interactions between its components, which can be influenced internally [26]. However, CAS are also nested within other systems and may be impacted by the external context in which it exists. The aim of this study was, therefore, to understand how external forces on the CAS disrupt fair judgement emerging.

## Methods

### Theoretical underpinnings

We employed reflexivity throughout the research process and it is described through the dimensions of ‘personal’, ‘interpersonal’, ‘methodological’ and ‘contextual’ [27]. Our research team consists of experienced health professions education researchers and clinicians, all familiar with the study content, having undertaken previous studies on fairness in assessment. As a research team we work in diverse contexts, representing a range of specialties and medical education research environments across different continents. LS has previously been involved in education in the Netherlands, however NV (who conducted the focus groups) was not previously known to the participants. This diversity of experiences was leveraged through allowing for a range of perspectives and enabling rich team discussions during data interpretation [28].

Barriers and enablers often, have a real and realist aspect. In this study, however, we wanted to focus on limiting and enabling forces as partially constructed by participants in line with our constructionist paradigm [29]. For the rest of the paper, for readability, barriers and enablers will be referred to as forces. Specifically, we used principles of complexity theory as a ‘lens’ to identify how external forces may facilitate or disrupt fairness in assessment [30]. We recognise that complexity science is not singular, but rather has multiplicity of legitimate orientations [10]. Our focus was on how individual elements work in concert to create systems with complex behaviour. This approach to complexity lends itself to social phenomena and is commonly used in medical education. Whilst there are many legitimate approaches to and principles of a CAS, we have used three of the key principles, interaction, adaption and embeddedness to identify how external forces may impact fairness [6,8,12]. The first two were chosen because it is only through interaction and adaption of individual elements that diverse behaviour or outcomes can emerge from the CAS [12,31]. In other words, the whole is more than the sum of the parts. Embeddedness was chosen because the individual agents and systems also sit within other systems. Medical schools, for example are embedded within clinical workplaces, which are embedded within society. CASs cannot be fully understood without reference to these other systems [11,14]. In addition, we have defined three subcategories of forces (assessor and student, tool and system forces) to follow the three components described by Schuwirth and van der Vleuten in their description of the history assessment [2]. In this paper, assessment was described as a measurement (tool), judgement (assessors and students involved in the judgement) and system. Specifically, a ‘tool’ is information, assessment or any strategy which would normally support interactions to facilitate the emergence of fairness.

### Setting and participants

This study was conducted online via Zoom with a purposeful sample of academic leaders from eight universities across the Netherlands. All universities involved in this research were either utilising or transitioning to a programmatic assessment framework. The Netherlands also has a thriving, collaborative medical education community and participants would be well informed to discuss this topic with a good understanding of the literature. Medical training within the Netherlands involves a three-year Bachelor of Medicine programme followed by a three-year Masters of Medicine programme [1]. All eight medical schools were invited to participate through the Dutch Association for Medical Education. Each medical school was able to nominate appropriate individual members to participate. Ethics approval was obtained (Flinders University: 4297).

We invited participants via email to participate in focus groups. Focus groups were chosen to allow individuals to build on other group members’ responses, allowing for dynamic interactions [32]. As we wished to understand the external forces on the CAS which may impact fair judgement emerging, participants were shown a video explaining the findings of our related series of studies. In the focus groups, we asked them to discuss their perspectives on considering fairness as a complex adaptative system, as well as the external systems or factors which could influence the CAS. We provided no further incentive to participate.

### Data analysis

The focus groups were recorded and transcribed verbatim without any identifying data. Reflective notes collected during focus groups and the shared white boards were included in data analysis. We used NVivo (Denver, Colorado) qualitative software to assist with data management. Although there are elements of abductive analysis, most of the first and second order themes were already preconceived and so this methodology is best described as thematic analysis. As data collection progressed, we developed codes, and refined and revised them in an iterative matter. The analysis process involved development of a coding book, comparison of different codes between and within transcripts to clarify, confirm and categorise codes. Themes were then developed and illustrative quotes were used to bring the participant experiences to light. All authors were involved in the discussions during the coding process. The data collected was considered to offer a sufficient understanding (drawn from Dey’s notion of theoretical sufficiency) to answer the research question [28].

## Results

Four focus groups were held between February and March 2022 lasting between 70–95 minutes. Nineteen individuals from six medical schools participated. As described above, there are forces which can impair interactivity, adaptability and embeddedness and restrict fairness from emerging. Within these themes, the forces have been subcategorised as assessor and students forces, tool forces and system forces (Table 2).

**Table 2:** The forces preventing fairness emerging from the complex adaptive system.


“COMPLEX”: FORCES IMPAIRING INTERACTIVITY

**Assessor and student forces** Assessors’ enthusiasm and engagement in the judgements process Assessor self-doubt and lack of confidence in their own judgement Student not empowered to interact with the complex adaptive system Student chooses not to engage Lack of situational awareness

**Tool forces** Not using evidence or tools to mediate interactions Use of convenience not purposeful sampling to support interactions Lacking information to support meaningful interactions Lack of access to information

**System forces** System barriers, hierarchical systems and cultural norms can inhibit opportunities for interactions between stakeholders

**“ADAPTIVE”: FORCES IMPAIRING ADAPTABILITY**

**Assessor and student forces** Assessor inexperience which impacts their ability to adapt in response to their interactions Assessors not adapting due to fear of change and uncertainty, or of doing wrong Assessors not appreciating need to adapt (I know best) or not wanting to adapt (easier not to) Learners are unaware of how to interact and adapt with the judgement Learners unwillingness to adapt following negative feedback Learners inappropriately adapt their behaviour towards those assessing them to receive a desired outcome

**Tool forces** Articulation of judgement to facilitate adaption Willingness of assessors to give and receive feedback to each other

**System forces** System which does not allow for feedback and adaption

**“SYSTEM”: FORCES IMPAIRING EMBEDDEDNESS**

**Assessor and student forces** Unsafe for a learner to be vulnerable to judgements Vulnerability of assessors as they have ultimate responsibility for their patients Lack of support for assessor to make a judgement

**Tool forces** High stakes nature impacts perception of fair

**System forces** Judgements influenced by bias, such as gender bias, harmful discrimination or specific prejudices which are outside agreed fuzzy boundaries Conflict in the purpose of judgement for the individual: is it a progression judgement or feedback? Conflict in the purpose of judgement for the system: it is distinguishing between learners, ie ranking or determining if meeting a standard? Fear of an external force which will disrupt the system University regulations limit the freedom of assessors to make judgement decisions System ensures some judgements are more intensive than others, ie fail judgements University limitations (ie high student numbers, money, assessor time, inefficient technology) impact how assessors interact with the system


### Forces impairing interactivity

A fundamental characteristic of a CAS is that the system’s behaviour relies less on the nature of the individual agents than on the quantity and quality of the interactions between them. Barriers to these interactions can cause significant disruption the output of the system.

#### Assessor and student forces

Assessors can self-limit their interactions with the components of fairness, for example as a result of their self-doubt in making a judgement, limiting the emergence of fair judgement. Their self-doubt may be in their own abilities, or it may be due to concern there is a lack of information to form comprehensive picture of a learner’s progress. *“Confidence in their own judgement … People doubt whether they really have all the reason(s) to give this nice person the judgement you’re not good enough at this moment.”* (Participant 1)

On the other hand, enthusiasm and engagement of assessors, or perception of engagement, in the assessment judgement process impacts the quantity and quality of these interactions. This may be through permitting or not empowering the learner to be an active participant in the learning process, or because the student chooses not to engage. Either way, the level of engagement may impact on the intent of wanting students to learn and improve, which directly influences the perception of fairness from both an assessor and learner perspective. *“These often are also students that don’t have ownership of their portfolio. They don’t own their learning path.”* (Participant 6)

Provision of feedback to the student requires situational awareness and meaning-making of the situation in the here and now. Unless this meaning-making, situational awareness and agile adjustment of behaviour occurs, interactions will be limited. Typically, the process is likely to be perceived as ritualistic and going through the motions.

*“She [student] said, in the one internship I got the command that I wasn’t assertive enough, I didn’t speak up enough, so I tried to change that in the next internship, and then they told me I did too much. I spoke too much, and spoke up too much. I was too dominant, I was – so, actually, I don’t know any more what I should do.”* (Participant 11)

This is also an example of gender bias which is described later. If an assessor’s judgement is influenced by any factor other than the student’s performance, then this is outside of the agreed fuzzy boundaries and a pressure on the CAS. This includes biases, stigma or harmful discrimination.

#### Tool forces

Having sufficient information about the learner facilitates interactions. So, logically, barriers to the provision and availability of information limits interactions. Examples of this include assessors not making the effort to collect sufficient information to support interactions, *“…many teachers [sic] do not look in the portfolio”* (Participant 18) or only using evidence that is convenient to find, *“…not use like convenience sampling, like the patients that are just coming along, and also use like more purposeful sampling that you say okay, we’re missing your data on your ability to handle such kind of patients”* (Participant 2) or not being able to obtain the meaningful information, that is needed to support these interactions. *“the system doesn’t provide this type of feedback because they did a multiple-choice test.”* (Participant 7)

#### System forces

Hierarchical systems and cultural norms may limit interactions between assessors and students. For example, learners with different backgrounds and cultural differences may face difficulties in adopting to assessment within their new system. If their cultural norms do not align with assessment process this can limit their ability to engage with the system. Traditional beliefs about the value proposition or role of assessment in education can also discourage dialogue about assessment or feedback. This can further be complicated by system forces such as scheduling which can also limit the opportunities for interactions to occur both between assessor and student, and also between assessors. *“It’s a conservative, hierarchic system and a lot of surgeons, especially in our region, they’re not raised there, they didn’t have their education there, … so they’re not used to this kind of assessing, and they just think you shut up and you do your job.”* (Participant 7)

### Forces impairing adaptability

Assessors in fair assessment processes agilely adapt to past experience, internal and external influences and feedback. This makes a CAS efficient and effective because assessors use their expertise and situational awareness to quickly adjust their behaviour and interactions when necessary. Learners also contribute to this process of adaption. Any barrier to the adaptive processes will impact the behaviour of the system.

#### Assessor and student forces

Assessors may lack the expertise and situational awareness required to adapt to the incoming feedback and changing contexts, for example as a result of insufficient staff development. This increases the likelihood of a fear of change or intolerance of uncertainty, leading assessors to want to stick with ‘what they have always done’. But this comes with a lack of perspective as to the need to adapt, or not wanting to adapt, or even the belief that everything was better in the past. Such a misalignment between what the assessor has to offer and what the situation at hand requires, easily leads to a perception of unfairness.

*“We know that teachers have certain conceptions of learning, which have been formed by their own experience, and those conceptions of learning – and assessment – are deeply rooted, and it’s very hard to change them…They’re often also they’re formed by their personal experience, and perhaps even related to their identity as a teacher.”* (Participant 12)

Assessors may also fear they are not acting in the best interest of both students and society, so do not adapt to avoid doing the ‘wrong’ thing; or that formative and summative assessment or assessment of and assessment for learning are zero-sum games. *“…they [assessors] want to do the best thing, and they’re afraid that with the new way of assessing, these programmatic assessments, they are afraid that they are not doing the right thing”* (Participant 13)

Assessors may fear students too may be uncertain about how to interact and adapt to the judgments and that they see it as a zero-sum game as well. This, again, may impact on the adaptability of stakeholders in the CAS and limit the emergence of fairness. *“…in a summative system, we sort of educate learners to ignore feedback”* (Participant 12)

This may be due to many reasons such as cultural values, previous experience with assessment, or expectations placed on doctors within society to not show weakness and thus not need to adapt. *“…one of my major goals in life is if I can achieve that doctors consider it normal to be vulnerable, I would think that we have gained a lot. But there are still many around who feel themselves or think they are still on this pedestal, and they can’t make any mistakes.”* (Participant 17)

Students may be unwilling to adapt due to the stigma or embarrassment of receiving negative feedback which may hinder future interactions in the system. The student may identify reasons for receiving this negative feedback, including being a surprise result, or it being the fault of an unfamiliar way of testing, but either way an unwillingness to adapt and learn from the negative feedback remains a barrier to the CAS. *“What I often see is that students who fail the test will say that the test was subjective. So, it was not their fault, it was the test’s fault. It’s used as a mechanism to not be open to learn”* (Participant 3)

Students may inappropriately adapt their behaviour towards those assessing them to receive a desired outcome. Both the assessor and student are behaving in a way in which means they are complying with the system but are self-limiting the quality of interactions because they don’t want to play the ‘real’ game of vulnerable, in-the-moment authentic learning and feedback. This may hinder the quality of further interactions.

#### Tool forces

Students and assessors are only able to adapt to enable fairness to occur if they receive information from stakeholder interactions. If information is not provided either because it is difficult to articulate or because assessors are unwilling to share, then purposeful adaption cannot occur. *“…a student can tick the boxes but you have the feeling that it’s not going to be a good doctor and how do you make that visual, visualise that to other teachers.”* (Participant 4) This includes interactions both between student and assessor and between assessors. *“…the openness to feedback and to give feedback to your fellow assessors.”* (Participant 2)

#### System forces

A system which makes stakeholders feel unsafe in providing feedback and engaging in interactions will not support interactions and adaptions. *“Someone [whistle-blower] who’s leaking information about certain circumstances, but they are the people who don’t feel they can be – they are not free to be honest.”* (Participant 13)

### Forces impairing embeddedness

Individual agents and systems are embedded within wider systems. Each individual or system cannot be fully understood without reference to their roles within wider systems.

#### Assessor and student forces

The diversity of roles of both the student and assessor can create barriers with the CAS. Students are not just learners, they are also future doctors attempting to obtain a grade, a residency training position or impress a future colleague. This variety of future roles within a variety of systems can make it unsafe for them to be vulnerable, engage in quality interactions and adapt appropriately to the judgement decisions. *“They all feel like they can’t show what they have in themselves, they can’t show their talents and they’re very worried that they will not get the job that they want so much and so on. It’s really a lot of tension actually.”* (Participant 15)

Similarly, assessors are not just assessors. They are also clinicians with responsibility for patients or in private practice, even business owners. Assessors may feel vulnerable and unable to trust learners with patient care. A limited perception of this entrustment hinders interactions in the workplace. When, for example, a learner feels that they could have been entrusted more than the assessor, this creates the perception of unfairness. *“I think they also feel vulnerable, they just have to let their student go to their very ill patients and … they don’t get feedback themselves of the capability of the student and it’s very difficult to let loose of that control.”* (Participant 15)

Assessors also work with the learners themselves, and need support to make judgement decisions, especially difficult judgement decisions.

#### Tool forces

Judgement decisions usually have wide-reaching effects. Some high stakes decisions can have significant financial, social or motivation consequences, especially if the judgement is that the learner is unsatisfactory or not ready to progress to a next phase. Tools that do not provide sufficient information to form such high-stakes decisions and require decisions that are not proportional with the richness of information available will be perceived as not fair and may evoke volatile emotional responses. These, in turn, will impact on future interaction with others and the system, such as leading to leniency bias or retreatism. *“But the two or three per cent of the students which will get an unsatisfactory grade, they say it’s not fair. It’s always the same [problem].”* (Participant 7)

#### System forces

Complexity thinking maintains that systems are not synonymous with complete chaos and that they can be maintained by fuzzy, ill-defined boundaries. If a judgement is influenced by any factor other than the student’s performance, then this was seen as being outside of these agreed boundaries and a pressure on the CAS. This includes biases, stigma or harmful discrimination.

*“…sometimes students are discussed in the staff meeting or someone will tell you about a student and I think that also influences your judgement. That can be particularly detrimental for what we call non-traditional students, so for people with migration backgrounds with lower socio-economic status.”* (Participant 2)

As the system is not isolated but rather overlaps with other systems, there may be conflicts between the various purposes of the judgements. This can put pressure on the system and its ability to adapt and future interactions. *“Am I giving feedback or I’m also giving a judgment?”* (Participant 14)

An external force, such as the COVID pandemic, or fear of an external force such as litigation is also likely to put pressure on the system. University level regulations tend to limit the freedom of assessors to interact, adapt and make judgement decisions and can make some judgement decisions more time consuming than other decisions. *“it also depends on the system, because I still can have the courage but I still can’t do it because of the system.”* (Participant 2)

Institutions also make decisions about how to spend finite resources which impact how assessors are able to interact with the system. *“You want to have a very individual, personal relationship, like a mentor, but for the start of the study, the bachelor part, numbers are too high. It’s very difficult.”* (Participant 13)

## Discussion

This study adds a different perspective on the external forces which may impact the emergence of fairness. Often barriers are described in realist terms; for example, lack of time or resourcing. However, this study uses the lens complexity to describe how forces prevent fairness from emerging from the CAS. Viewing forces in this way provides insight into why and how these forces are disrupting fairness from emerging. This is not trivial. When barriers and enablers are described in realist or objectivist terms it carries the connotation that they are relatively established. On the other hand, when barriers and enablers are explored from a subjectivist and complexity lens, it allows us to critically examine the factors which are contributing the creation and persistence of these forces, and agilely adapt or create more levers to influence the impact these forces have on the CAS and the emergence of fairness.

Typically, workplace-based assessment occurs in unpredictable clinical environments where implementation of replicable and standardised, measurement-based assessments is largely impossible. However, if we look beyond linear, objective thinking and a complexity lens is applied, then these challenges can be reconsidered. For example, they make us reconsider the value of equality versus the value of equity with respect to fairness. Equality, as in standardisation and structuring assessment may seem fair because everybody receives the same process of assessment. Our concept of fairness is one from an equity lens. Everybody receives the same quality of assessment, but the assessment process is bespoke; it recognises that people have different strengths and weaknesses, and that the assessment process needs to be bespoke to cater to those. Complexity thinking does not reduce the scale of the challenge, nor does it provide simple fixes to tensions in assessment [5]. But, it does provide a different perspective to approach them. It also presents forces as interactional problems which can be modified allowing institutions more agency over the situation.

Consistent with complexity theory, this study suggests there is no single force or factor which needs to be addressed for fairness to emerge. The study highlights an almost overwhelming number of potential forces to address. However, viewing these forces with a systems mindset has at least two important implications. Firstly, a systems mindset shifts responsibility from away the individual. Forces need to be addressed at a system level because forces arise from changing interrelationships or adaptions (or lack of) between parts of the systems [33]. Secondly, addressing this as a system, allows for a framework to allow the researcher and educator to better identify, explore and address the force and related potential forces.

The forces described in this study are not exhaustive; there are likely many others. Similarly, the generalisability of the forces identified is limited by the nature of this study. However, the intent of this inquiry was not to identify an exhaustive list, nor was it to design solutions as these too are likely to be context specific. The aim of this study was to understand the external forces which may impact the emergence of fairness using a lens of complexity. Considering fairness as a CAS changes our views about how we can improve assessment and legitimise human judgement in our assessment programs. Because in CAS, the interactions between the entities are most important, meaning strict regulatory frameworks and tick box approaches to managing fairness are counterproductive because they limit the interactions between components.

Embracing complexity in fair judgement also means understanding that managers or policies cannot control the judgements assessors make, or that linear, causal thinking cannot predict behaviour of the individuals in the system [13,14,34]. Instead, systems designs and management practices which encourage interactions, develop expertise, enable access to all necessary information, facilitate self-organisation and individual responsibility can contribute to better outcomes [7,35]. Providing a variety of strategies to enable assessors to adapt to the situation in the here and now rather than enforcing one ‘gold standard’ strategy is also likely to enhance system behaviour [8,36].

Our previous research into the components of fairness noted a fractal which consisted of credibility, fitness for purpose, transparency and accountability [26]. Fractals are shapes or concepts which exhibit “self-similarity” at different scales, meaning they remain the same regardless of whether you zoom in or out [37,38]. A fractal is a manifestation of an underlying complex adaptative system (CAS) [39]. Understanding fractal patterns can enable sense making in complex systems and guide rational changes in the system and influence the agent’s behaviour [9,12]. Fractals can provide structure and fuzzy boundaries to help CAS remain in stable equilibrium [8,9,34,38]. Because of the organised, adaptive nature of CAS, if any of the fractal elements are missing, the system becomes unstable and may breakdown [40]. This has implications for the way we design assessments.

There are limitations to this study. As already mentioned, the forces identified are not exhaustive; there are likely many others. Similarly, given the Western orientated cultural context, there may be additional relevant meaningful perspectives to be found in other contexts. Furthermore, whilst we specifically sought to look at forces from a constructionist perspective, researching forces from a realist perspective could complement this view, and may enable a more comprehensive approach to future system changes.

As 21^st^ century health professions education moves to embrace human judgement in its assessment programs [41] understanding what makes this judgement fair beyond an objective framework is essential. Understanding and thus modifying the forces which prevent fairness emerging in light of a CAS system can lead to more purposeful, meaningful changes to support the use of fair judgement in assessment in the authentic clinical workplaces.

## Additional File

The additional file for this article can be found as follows:

10.5334/pme.994.s1 Supplementary File. Focus group video script and question guide.
