# Supplementary material for: What Stops Fairness from Emerging in Assessment? The Forces on a Complex Adaptive System
Source: Perspect Med Educ. 2023 Aug 24;12(1):338–47. doi: 10.5334/pme.994 (PMC10453954; doi:10.5334/pme.994)
Supplement: Supplementary File. — Focus group video script and question guide. [file pme-12-1-994-s1.pdf]

APPENDIX 1:

Focus Group Video Script and Question Guide:

1 Most people agree that assessment needs to be fair. Traditionally, objectivity was seen as the main way to ensure fairness in assessment. But more recently, views have changed, and it is now generally accepted that subjective human judgement plays an key role in comprehensive assessment programs. However, in embracing subjective judgement an important question has arisen, what makes human judgement in assessment fair?

2 That is what we have been looking at with a series of studies.

SLIDE TRANSITION

And what we've found is that fair judgement in assessment is complex. It can actually be considered to be a complex adaptive system. As such, there are many interacting and sometimes conflicting factors to consider and understand.

SLIDE TRANSITION

3 To help explain what we've found, let's use an analogy. Consider a pine tree. A pine tree is composed of branches which are composed of smaller branches which in turn are composed of even smaller branches and so on. Branches on pine trees have an interesting feature: no matter where you look, or how much you zoom in or zoom out, the shape or pattern remains the roughly the same. From the largest branch to the smallest branch the pattern seems to repeat, over and over again at different scales. This is called a fractal. Fractals can also be produced mathematically. The equation behind repeating fractals is actually quite simple, but it produces an incredibly complex shape which repeats for infinity.

SLIDE TRANSITION

4 We think fair judgement in assessment is a little like this. It is complex and seems to be different in different circumstances. But if you look more closely, our research has demonstrated that there is a recurrent and repetitive shape to fair judgement. We've conducted a literature review, spoken with learners, teachers and education designers and managers. And what we found was that underlying everything they said were the same four components of fair judgement: transparency, accountability, fitness for purpose and credibility. This is the basic "shape" of fair judgement.

SLIDE TRANSITION

5 Just like the equations in fractals, these four components of fair judgement are reasonably straightforward in themselves. However, these components are not enough to create fairness in judgements on their own. Fair judgement 'emerges' from the purposeful and meaningful interactions between these four components. And it is these interactions which makes fair judgement complex. To use another analogy, when you take all of the components of the human body and put them into a bucket that does not create life. Life only exists when all of those body systems work together and interact with one another.

SLIDE TRANSITION

6 And there are many layers or sizes of pine branches. In fact, there is an almost infinite number of sizes that this same complex shape can be. The same is true of fair judgement. There are an infinite number of layers of judgement, for example, an individual utterance of the learner during the assessment, whether they were able to take a history from a patient, right the way through to is a learner ready to graduate? It doesn't make a difference if you look up closely, ie as an on the ground supervisor, or

take a step back as a program coordinator, in all of the layers the same four components of fair judgement can be seen.

SLIDE TRANSITION

7 There are also forces which influence the development of the complex shapes. Going back to our tree analogy, the growth and size of the pine tree is influenced by the sunlight, or soil quality or water. And if someone builds a great big building next to the pine tree, the shape is going to be altered too.

SLIDE TRANSITION

8 Similarly in fair judgement, there are forces which influence these four key components. These forces include being able to have multiple assessors, whether longitudinal data collection is possible, having a narrative or vocabulary to support the judgment and so on. These are demonstrated in the diagram provided. It is these forces which influence the interactions and linkages between the four components of fair judgement.

9 Our model demonstrates how we see the complex adaptive system of fairness in assessment. There might be other components but this is a framework to help understand and construct fair judgement in different contexts.

SLIDE TRANSITION

We do know though that know that fairness does not operate in a vacuum. It is impacted by various other systems and forces, for example university regulations, patient demands or power imbalances between assessors and learners. It is these other systems and forces, and how they impact on fair judgement that we are interested in for this study.

10 We'd love to know 3 things:

- What do you think of the model? We will go through this at the beginning of the focus group.
- What external systems or factors could influence our model?
- How do these external systems or factors influence the interactions between the elements of our model?
